# Three-Year Clinical Outcomes of Lithium Disilicate and Lithium-Silicate-Modified Zirconia Veneers and Partial-Coverage Restorations: A Retrospective Comparative Study

**DOI:** 10.3390/dj14100621

**Published:** 2026-09-26

**Authors:** Alexandra Cristina Măroiu, Andrei-Bogdan Faur, Andra Ardelean, Marius Leretter, Anca Jivănescu

**Affiliations:** 1 Department of Prosthodontics, University of Medicine and Pharmacy “Victor Babes”, B-dul Revolutiei 1989, No. 9, 300580 Timisoara, Romania; maroiu.alexandra@umft.ro (A.C.M.); andrei.faur@umft.ro (A.-B.F.); jivanescu.anca@umft.ro (A.J.); 2 TADERP Research Center, Department of Prosthodontics, University of Medicine and Pharmacy “Victor Babes”, B-dul Revolutiei 1989, No. 9, 300580 Timisoara, Romania; 3 University Clinic of Dentofacial Aesthetics, Faculty of Dental Medicine, University of Medicine and Pharmacy “Victor Babes”, B-dul Revolutiei 1989, No. 9, 300580 Timisoara, Romania

**Keywords:** ceramic veneers, partial-coverage restorations, lithium disilicate, zirconia, lithium silicate surface modification, Biomic LiSi Connect, adhesive dentistry, debonding, preparation design, clinical performance, retrospective study

## Abstract

**Background:** Clinical evidence regarding lithium-silicate-modified zirconia veneers and partial-coverage restorations remains limited. This retrospective study compared the observed three-year outcomes of two complete adhesive restorative protocols rather than the isolated effects of ceramic materials. **Materials and Methods:** One hundred ceramic veneers and partial-coverage restorations placed in eleven patients were evaluated after a three-year follow-up. Fifty restorations were fabricated from lithium disilicate (IPS e.max Press; Ivoclar Vivadent AG, Schaan, Liechtenstein), and fifty were fabricated from zirconia (Aidite Biomedical Co., Ltd., Qinhuangdao, China) treated with the Biomic LiSi Connect system. No patient received both protocols. Clinical records, photographic documentation, and recall examinations were reviewed for fractures, chipping, marginal discoloration, surface wear, secondary caries, hypersensitivity, repairs, replacements, and debonding. Outcomes were analyzed descriptively because multiple restorations were clustered within patients and protocol allocation followed clinical indication. Wilson 95% confidence intervals (CIs) were reported for restoration-level proportions as descriptive measures and were not adjusted for clustering. **Results:** Both protocols showed 100% survival without replacement (95% CI: 92.9–100.0%). Debonding affected 6/50 zirconia restorations (12%; 95% CI: 5.6–23.8%) and 0/50 lithium disilicate restorations (0%; 95% CI: 0.0–7.1%). The six events occurred in five of seven zirconia patients and none of four lithium disilicate patients. All debonded restorations were recemented and remained functional. No fractures, chipping events, repairs, or replacements were recorded. **Conclusions:** No replacement or structural failure was observed during three years in this selected convenience cohort. However, the zirconia protocol had six retention-related interventions. Because the protocols differed in material, surface treatment, cement, indication, and anatomical distribution, the findings are exploratory and do not establish an isolated material effect.

## 1. Introduction

The growing emphasis on tooth preservation has strongly influenced the planning of indirect ceramic restorations. In the anterior region, ceramic laminate veneers allow modification of tooth color, shape, and alignment with minimal removal of sound tooth structure. Similar minimally invasive principles have been extended to posterior teeth through adhesively bonded onlays, overlays, and occlusal veneers. Although these restorations differ in indication and geometry, they can be considered within the broader category of partial-coverage ceramic restorations because they replace only the damaged, deficient, or aesthetically compromised portion of the clinical crown [1,2].

The clinical success of ceramic laminate veneers is well documented. In a systematic review, Morimoto et al. reported high survival rates for feldspathic porcelain and glass-ceramic laminate veneers, although fractures, debonding, marginal discoloration, and secondary caries remained the main complications observed during follow-up [3]. More recently, Klein et al. reported survival rates exceeding 93% after approximately ten years for feldspathic, leucite-reinforced, and lithium disilicate veneers [4]. Among the investigated glass ceramics, lithium disilicate showed a survival rate of 96.81% and fewer technical complications [4].

Additional clinical studies have confirmed the favorable performance of lithium disilicate restorations. A ten-year retrospective study of heat-pressed lithium disilicate veneers reported a survival rate of 97.4% [5], while a clinical evaluation of 1075 CAD/CAM lithium disilicate veneers found a cumulative survival rate of 99.83% over a mean follow-up period of 30.8 months [6]. In posterior rehabilitation, adhesively bonded lithium disilicate occlusal onlays have also demonstrated excellent clinical performance, with 100% survival reported for up to 11 years [7].

Lithium disilicate is widely used for partial-coverage restorations because it combines favorable optical properties, adequate mechanical strength, and a predictable bonding protocol. Hydrofluoric acid selectively etches the silica-rich glassy phase, creating a micromechanical surface that can interact with silane coupling agents and resin cement [8].

When sufficient enamel is available for bonding, the ceramic restoration, adhesive interface, and tooth structure function as a reinforced biomechanical complex. Enamel preservation is therefore central to minimally invasive restorative dentistry and is clinically relevant for restoration longevity. Higher failure rates have been reported when laminate veneers are bonded predominantly to dentin or composite restorations rather than enamel [9].

Despite these advantages, lithium disilicate may not provide sufficient masking in all clinical situations. Highly discolored teeth, endodontically treated teeth with dark substrates, metal posts or core build-ups, and pronounced substrate color discrepancies may require either increased ceramic thickness or an opaquer restorative material. Increasing the thickness of a glass-ceramic restoration can compromise the objective of minimizing tooth reduction, creating a clinical need for alternative materials in selected cases.

Advances in zirconia composition and processing have expanded its potential use in such situations. Newer translucent zirconia materials offer improved optical properties and can be fabricated in relatively thin sections, making them suitable for selected anterior veneers and posterior partial-coverage restorations [10]. However, increased translucency in Zirconia is associated with changes in its chemical and physical properties, including potential effects on transformation toughening [10].

The clinical performance of zirconia partial-coverage restorations cannot be evaluated on the basis of mechanical strength alone. In the systematic review by Klein et al., zirconia laminate veneers showed promising short-term results, but the available literature included substantially shorter follow-up periods than those reported for lithium disilicate veneers [4]. Consequently, the medium- and long-term behavior of zirconia veneers and partial-coverage restorations, particularly their adhesive retention, remains insufficiently documented.

The main limitation of zirconia in adhesively bonded partial-coverage restorations is the adhesive interface. Unlike lithium disilicate, zirconia is a polycrystalline ceramic without a silica-rich glassy phase and is therefore not predictably conditioned by the conventional hydrofluoric acid-silane protocol.

Bonding to zirconia relies on mechanical surface modification, such as airborne particle abrasion, chemical interaction with phosphate-containing monomers such as 10-methacryloyloxydecyl dihydrogen phosphate (10-MDP), or combinations of surface treatment and functional primers [11,12,13,14]. Although these approaches can improve initial bond strength, the durability of the resin-zirconia interface may be affected by hydrolytic degradation, surface contamination, air-abrasion parameters, zirconia composition, and primer-cement compatibility [11,12,13,14].

One proposed strategy for overcoming these limitations is surface modification of the zirconia intaglio surface with silica- or lithium silicate-containing coatings. After thermal treatment, such coatings create a glass-ceramic-like surface that can be etched with hydrofluoric acid and silanized, thereby applying a more established glass-ceramic bonding concept to a difficult-to-condition substrate. Laboratory studies have shown that lithium disilicate-containing intermediate layers may influence bond strength and failure mode in bilayered zirconia systems; however, the magnitude of this effect depends strongly on material properties, coating thickness, treatment protocol, and testing conditions [15]. Moreover, favorable laboratory bond-strength values do not necessarily predict clinical longevity because adhesive joints are exposed intraorally to moisture, temperature changes, fatigue loading, and variation in tooth substrates [16].

The Biomic LiSi Connect system is designed to create a lithium silicate layer on zirconia before adhesive cementation. From a clinical perspective, this approach is attractive because it aims to combine the strength and masking ability of zirconia with a modified intaglio surface that resembles an etchable glass ceramic. However, it remains unclear whether zirconia restorations treated with this system can achieve retention comparable to that of conventionally etched lithium disilicate restorations. Preparation geometry may also influence restoration retention. Although enamel preservation is essential, bonding surface area and functional stress distribution are affected by the extent of tooth preparation.

The exact date of commercial introduction of Biomic LiSi Connect could not be independently verified from the sources available to the authors. Accordingly, the system is described as relatively recent, and the limited independent medium- and long-term clinical evidence is acknowledged explicitly.

Proximal wrap-around, contact-area removal, incisal extension, and partial or complete occlusal coverage can alter both the available adhesive surface and the pathway through which functional loads are transferred from the restoration to the tooth. These variables may be particularly relevant for zirconia restorations because their adhesive interface remains more technique-sensitive than that of silica-based ceramics. However, the relationship between preparation geometry and debonding of zirconia partial-coverage restorations is not well understood.

The available evidence concerning lithium-silicate-containing or glass-ceramic surface modification of zirconia is predominantly laboratory-based. Medium-term clinical data remain limited. The primary objective of this retrospective study was therefore to compare the three-year observed outcomes of two complete adhesive restorative protocols: lithium disilicate restorations and zirconia restorations treated with a manufacturer-designed lithium-silicate-containing surface-modification system. Particular attention was paid to retention-related complications.

The distribution of debonding according to preparation design was explored descriptively within the zirconia group; the study was not designed to isolate a causal material or coating effect.

## 2. Materials and Methods

### 2.1. Study Design

This retrospective comparative clinical study evaluated the three-year clinical performance of lithium disilicate and zirconia veneers and partial-coverage ceramic restorations. Zirconia restorations were treated with a lithium silicate surface-modification system (Biomic LiSi Connect; Aidite Biomedical Co., Ltd., Qinhuangdao, China) before adhesive cementation. The analysis was based on clinical records, photographic documentation, and recall examinations from patients treated in a private dental practice.

The primary objective was to compare the three-year observed outcomes of two complete adhesive restorative protocols: lithium disilicate restorations and zirconia restorations treated with Biomic LiSi Connect. Retention-related complications were of particular interest. The association between preparation design and debonding was explored descriptively within the zirconia group.

The study was conducted in accordance with the Declaration of Helsinki and was approved by the Ethical Committee of the University of Medicine and Pharmacy “Victor Babeș” of Timișoara, Romania. All included restorations had a documented three-year observation period. To avoid ambiguity, the follow-up interval was defined individually for each restoration as the period between definitive cementation and the three-year recall examination.

### 2.2. Patient Selection and Clinical Distribution

Patients were selected from the clinical database according to predefined eligibility criteria. Only cases with complete clinical documentation, photographic records, and a documented three-year follow-up after definitive cementation were eligible for inclusion.

Inclusion criteria were as follows: ceramic veneers or partial-coverage ceramic restorations fabricated from lithium disilicate or zirconia; complete clinical and photographic documentation; documented three-year follow-up after definitive cementation; and availability of periodic recall examinations.

Exclusion criteria were as follows: incomplete clinical records; absence of standardized photographic documentation; follow-up shorter than three years; loss to follow-up; or restoration replacement for reasons unrelated to material performance.

Restorations were included only when definitive cementation and the three-year recall were documented. Case selection operated first at the patient level. All 50 available lithium disilicate restorations from four eligible patients were included. Seven eligible zirconia patients had more restorations available than required for the prespecified descriptive comparison; 50 zirconia restorations were selected to retain contributions from all seven patients and representation of both arches and anterior and posterior regions. This availability-based, non-random selection is a potential source of selection bias (Figure 1).

A total of eleven patients were included: four received the lithium disilicate protocol and seven received the zirconia protocol. No patient received both restorative protocols. The study population consisted of seven female and four male patients, with a mean age of 42.4 ± 7.1 years (range: 32–54 years).

The final descriptive sample comprised 100 ceramic veneers and partial-coverage restorations: 50 lithium disilicate restorations contributed by four patients and 50 zirconia restorations treated with Biomic LiSi Connect contributed by seven patients. Because the study was retrospective, no prospective sample-size calculation was performed.

The patient was the independent clinical cluster, and the restoration was the unit used to record local complications. Multiple restorations within the same patient were not considered statistically independent. Patient-reported outcomes were evaluated at the patient level. Table 1 reports the number of restorations and debonding events contributed by each anonymized patient.

The anonymized restoration-level dataset, including patient ID, material protocol, tooth, arch, region, preparation design, and debonding status, is provided in Supplementary Table S1.

Equal numbers of restorations were available for descriptive comparison; however, protocol allocation was determined by clinical indication rather than randomization. Of the 100 restorations, 58 were maxillary and 42 were mandibular. The selection process is summarized in Figure 1, and the anatomical distribution is presented in Table 2.

The zirconia group included a broader range of preparation designs because zirconia was used in more variable clinical indications, particularly where increased masking ability was required. Lithium disilicate restorations were more commonly used for conventional anterior veneer indications. This clinical heterogeneity was considered when interpreting the descriptive preparation-design analysis.

### 2.3. Study Groups

The included veneers and partial-coverage restorations were allocated into two groups according to restorative material.

Group 1: Lithium disilicate restorations.

Group 1 consisted of 50 restorations fabricated from lithium disilicate ceramic (IPS e.max Press; Ivoclar Vivadent AG, Schaan, Liechtenstein). Lithium disilicate was selected because of its well-documented clinical performance, favorable optical properties, and predictable adhesive protocol.

Group 2: Zirconia restorations with lithium silicate surface modification.

Group 2 consisted of 50 restorations fabricated from zirconia (Aidite Biomedical Co., Ltd., Qinhuangdao, China). Before adhesive cementation, the intaglio surfaces were treated with the Biomic LiSi Connect system, which is designed to create a lithium silicate surface layer and improve bonding to resin-based luting materials.

Zirconia restorations were primarily indicated in clinical situations requiring increased masking ability, including teeth with severe intrinsic discoloration or dark underlying substrates. The lithium silicate coating was applied to support adhesive cementation and clinical retention.

Both protocols included maxillary and mandibular, anterior and posterior restorations. The lithium disilicate group comprised 30 maxillary and 20 mandibular restorations; the zirconia group comprised 28 maxillary and 22 mandibular restorations. The groups differed in anatomical distribution and clinical indication, and all comparisons were therefore treated as exploratory.

### 2.4. Clinical Procedures

#### 2.4.1. Digital Treatment Planning and Mock-Up-Guided Workflow

Treatment planning incorporated a digital smile design and virtual wax-up. The digital design was transferred to a three-dimensional printed model and intraoral mock-up; subsequent clinical verification and adjustments were performed conventionally when required. The workflow should therefore be understood as digitally assisted rather than exclusively digital.

The virtual design was subsequently transferred into a three-dimensional printed model, which was used to fabricate an intraoral mock-up. The mock-up served as a diagnostic and communication tool, allowing both the clinician and the patient to visualize the anticipated treatment outcome before any irreversible procedures were initiated.

In addition to its diagnostic value, the mock-up was used to guide tooth preparation. This approach enabled a controlled and minimally invasive reduction in dental tissues, ensuring that tooth preparation was performed as conservatively as possible while providing sufficient restorative space and preserving the maximum amount of enamel.

The digital workflow and the sequence of treatment planning procedures are illustrated in Figure 2.

#### 2.4.2. Tooth Preparation

All tooth preparations were performed by the same clinician and were guided by the digital wax-up and intraoral mock-up. The protocol followed established minimally invasive veneer-preparation principles and was intended to provide the planned restorative space while preserving enamel whenever clinically possible [1,9].

The standard preparation protocol consisted of:

- Approximately 1.0 mm incisal reduction using a butt-joint finish line;
- Approximately 0.5 mm facial reduction;
- Proximal reduction of approximately 0.2–0.3 mm;
- A rounded shoulder or chamfer cervical finish line measuring approximately 0.3–0.5 mm and located predominantly at the supragingival level.

Although a standardized preparation protocol was followed, the extent of the proximal preparation was individualized according to the restorative objectives, initial tooth morphology, and the modifications required by the digital treatment plan.

Three preparation designs were identified according to the extension of the preparation on the proximal surfaces:

Type I: proximal extension involving approximately one-half of the mesial and distal surfaces without elimination of the contact points.

Type II: preparation extending over the entire proximal surfaces up to the palatal aspect without elimination of the contact points.

Type III: preparation extending over the entire proximal surfaces up to the palatal aspect with elimination of the contact points. This design was employed when modifications in tooth morphology, closure of interdental spaces, replacement of existing composite restorations, or substantial esthetic corrections were required.

For posterior restorations, additional preparation designs were used according to the amount of occlusal coverage required by the restorative plan. Partial occlusal coverage consisted of preparation extending to the mesiodistal occlusal groove or involving approximately one-half of the occlusal table, whereas complete occlusal coverage involved preparation of the entire occlusal surface. These designs were applied exclusively in posterior teeth when additional restorative space or reinforcement of the restoration was considered necessary.

Representative examples of the three preparation designs are illustrated in Figure 3.

Tooth preparation was performed using a standardized set of diamond burs (Komet Dental, Lemgo, Germany), including depth-cutting burs for initial reduction, cylindrical coarse-grained burs for shaping, and fine-grained finishing burs for surface refinement. Final smoothing was performed using polishing instruments to obtain a uniform and homogeneous preparation surface.

#### 2.4.3. Laboratory Procedures

All restorations were fabricated by experienced dental technicians according to standardized laboratory protocols.

Lithium disilicate veneers were manufactured using the heat-press technique (IPS e.max Press; Ivoclar Vivadent AG, Schaan, Liechtenstein) following the manufacturer’s recommendations. Zirconia veneers were fabricated using CAD/CAM technology from zirconia blanks (Aidite Biomedical Co., Ltd., Qinhuangdao, China). Following digital design, the zirconia restorations were milled, sintered, and finished according to the manufacturer’s protocols before adhesive surface treatment and cementation.

The morphology, contours, and occlusal relationships of all restorations were verified before final delivery. Cervical marginal adaptation and integration of the restorations within the planned smile design were carefully evaluated.

Representative examples of digital simulations and definitive restorations are illustrated in Figure 4.

#### 2.4.4. Surface Treatment and Adhesive Protocol

The adhesive cementation procedure was standardized for both restorative groups. The principal differences between groups consisted of the surface conditioning protocol applied to the restoration intaglio surface and the type of resin cement used for definitive cementation.

For lithium disilicate veneers, the intaglio surfaces were etched with 5% hydrofluoric acid (IPS Ceramic Etching Gel; Ivoclar Vivadent AG, Schaan, Liechtenstein) for 20 s, thoroughly rinsed, and subsequently cleaned in an ultrasonic bath to remove residual contaminants. A silane coupling agent (Monobond Plus; Ivoclar Vivadent AG, Schaan, Liechtenstein) was then applied for 60 s according to the manufacturer’s recommendations.

For zirconia restorations, Biomic LiSi Connect (Aidite Biomedical Co., Ltd.) was applied in the dental laboratory as a manufacturer-designed lithium-silicate-containing surface-modification protocol and thermally treated according to the manufacturer’s instructions. The exact Aidite zirconia trade name, yttria content, batch number, restoration thickness range, and case-specific firing records were not retained in the retrospective clinical database and could not be reconstructed reliably. After thermal treatment, the modified intaglio surface was air-abraded with 50-μm aluminum oxide particles in a Renfert laboratory unit for approximately 1 s at an approximate working distance of 30 mm. Air pressure, application angle, and suction settings were not recorded. The surface was then etched with 5% hydrofluoric acid for 20 s, rinsed, ultrasonically cleaned, and treated with Monobond Plus for 60 s. The intended mechanism is described according to manufacturer documentation; no chemical or microscopic characterization was performed in this study.

The prepared dental surfaces were etched with 37% phosphoric acid for 20 s, thoroughly rinsed, and gently air-dried. A universal adhesive system (Adhese Universal; Ivoclar Vivadent AG, Schaan, Liechtenstein) was subsequently applied according to the manufacturer’s instructions.

For restorations cemented using light-cured resin cement, the adhesive layer was gently air-thinned and left uncured. For restorations cemented using dual-cured resin cement, the adhesive layer was light-polymerized for 20 s before restoration placement.

Lithium disilicate restorations were cemented using a light-cured resin cement (Variolink Esthetic LC; Ivoclar Vivadent AG, Schaan, Liechtenstein), whereas zirconia restorations were cemented using a dual-cured resin cement (Variolink Esthetic DC; Ivoclar Vivadent AG, Schaan, Liechtenstein). The use of dual-cured cement for zirconia restorations was preferred because the increased opacity and thickness of zirconia may reduce light transmission and potentially compromise the polymerization of exclusively light-cured materials.

Following restoration seating, a short tack-curing cycle of approximately 2 s was performed to facilitate the removal of excess cement. Final polymerization was subsequently carried out for 60 s from both the facial and palatal aspects of each restoration.

In cases of zirconia veneer debonding, the restorations were reconditioned using the same Biomic LiSi Connect surface modification protocol and were recemented following the identical adhesive procedure described above. Residual adhesive and resin cement were removed from the dental substrate using a red-coded multi-bladed finishing bur. The dental surface was subsequently refined with an Arkansas stone and air-abraded before repeating the tooth-conditioning protocol described above.

Retention-related outcomes were emphasized because adhesive stability remains a recognized limitation of zirconia veneers and partial-coverage restorations. Biomic LiSi Connect was one component of the zirconia protocol. Because the study lacked untreated zirconia and alternative-conditioning controls, it could not determine the independent contribution of the coating, silane, micromechanical treatment, phosphate monomers, or resin cement.

#### 2.4.5. Occlusal Adjustment and Follow-Up

Following adhesive cementation, occlusal contacts were carefully evaluated in maximum intercuspation and during excursive movements. Whenever necessary, selective adjustments were performed to eliminate premature contacts and minimize excessive functional loading on the restorations.

All patients were enrolled in a maintenance program and were clinically monitored throughout the three-year follow-up period. Periodic recall examinations included professional prophylaxis and comprehensive clinical assessment of the restorations.

Clinical evaluations included the assessment of restoration retention, marginal adaptation, surface quality, color stability, postoperative sensitivity, periodontal response, and the presence of biological or mechanical complications. Veneer debonding and the clinical outcomes of recemented restorations were specifically monitored.

At the final three-year recall, patient satisfaction was assessed once using a study-specific 10-item questionnaire with a 0–10 Visual Analog Scale response format, where 0 represented complete dissatisfaction and 10 complete satisfactions [17]. The questionnaire was not a previously validated multidimensional patient-reported outcome instrument and was interpreted descriptively.

Standardized intraoral photographs and clinical records obtained during recall visits were subsequently reviewed for retrospective analysis. Any event requiring clinical intervention, including debonding, fracture, repair, recementation, or replacement of the restoration, was documented together with the corresponding management procedure.

Survival without replacement was defined as the restoration remaining in situ and functional at the three-year evaluation, with or without recementation. Debonding-free survival was considered separately. No restoration experienced repeated debonding; each of the six events involved a different restoration.

### 2.5. Outcome Measures

The clinical performance of all veneers and partial-coverage restorations was evaluated using predefined outcome measures. The assessed variables and their definitions are presented in Table 3.

Patient-reported outcomes were additionally assessed using a 10-item Visual Analog Scale (VAS) questionnaire. Each item was scored on a scale ranging from 0 (completely dissatisfied) to 10 (completely satisfied). The questionnaire evaluated the patients’ perceptions regarding the esthetic and functional outcomes of treatment, including overall satisfaction, smile appearance, tooth color, tooth shape, natural appearance, chewing comfort, speech and phonetics, integration of the restorations within the smile, gingival appearance, and willingness to undergo the same treatment again and recommend it to others. The evaluated domains are summarized in Table 4.

A composite complication-free outcome was not calculated because the retrospective records did not permit reliable determination of overlap among marginal discoloration, secondary caries, and hypersensitivity within individual restorations. Each outcome was therefore reported separately. Survival without replacement must not be interpreted as intervention-free or complication-free success.

Restoration survival was defined as the restoration being present in situ and functional at the end of the three-year follow-up period, regardless of whether recementation or other minor clinical intervention was required. This definition distinguished survival from complication-free success.

Debonding was considered the primary mechanical complication of interest and was analyzed separately because of its potential association with preparation design, restoration location, and adhesive surface extension.

### 2.6. Evaluation of Preparation Design and Debonding

Because debonding represented the most clinically relevant complication observed during the follow-up period, a dedicated analysis was performed to investigate potential factors associated with loss of restoration retention.

For each debonding event, clinical records, intraoral photographs, and treatment documentation were retrospectively reviewed. Preparation design, adhesive surface extension, restoration location, and occlusal characteristics were reviewed in detail.

Preparation designs were categorized according to proximal extension, management of proximal contact areas, and occlusal extension. Three anterior preparation configurations (Types I–III) and two posterior preparation designs (partial and complete occlusal coverage) were identified and descriptively analyzed.

Preparation extension was evaluated as a surrogate for potential bonding area; bonding surface area was not measured in square millimeters. The analysis therefore examined descriptive patterns according to preparation geometry and did not assume that greater extension directly represented a quantified increase in adhesive area.

In addition to preparation design, the anatomical location of the restoration and its position within the maxillary or mandibular arch were recorded for each debonding event in order to explore the potential influence of functional loading on restoration retention.

The results of this subanalysis were used to identify descriptive trends that may inform future clinical studies. No causal conclusions were drawn from this exploratory analysis.

When documentation allowed, the preparation design associated with each debonding event was descriptively compared with clinically stable restorations to identify possible trends related to adhesive surface area and retention.

### 2.7. Statistical Analysis

Categorical variables were summarized as counts and percentages. Restoration-level proportions are accompanied by two-sided 95% Wilson confidence intervals as descriptive measures of precision. These intervals do not account for within-patient clustering and should therefore be interpreted cautiously. Continuous patient-reported outcomes were summarized as mean ± standard deviation at the patient level.

Patient-reported outcomes were analyzed at the patient level: four patients in the lithium disilicate group and seven patients in the zirconia group. No restoration-level inferential testing was performed.

Given the limited sample size and the exploratory nature of the study, the analyses focused primarily on descriptive comparisons between restorative systems and the identification of potential clinical trends associated with restoration performance and debonding events.

No inferential comparison, random-effects model, or cluster-robust regression was performed because only 11 patient clusters and six debonding events were available. A post-hoc power calculation treating the 100 restorations as independent was also not performed because it would over-state the effective sample size. Descriptive analyses were performed using IBM SPSS Statistics for Windows, Version 24.0 (IBM Corp., Armonk, NY, USA).

### 2.8. Survival Assessment

Restoration survival was evaluated for both restorative systems throughout the three-year follow-up period. A veneer or partial-coverage restoration was considered to be surviving if it remained functional and in situ at the end of the observation period, irrespective of whether recementation had been required.

Clinical failures were recorded according to the predefined outcome measures described in Section 2.5. Debonding events were analyzed in detail because loss of retention represented the primary complication observed during follow-up and constituted the principal difference between the two restorative groups.

For descriptive purposes, survival rates were calculated separately for lithium disilicate restorations and zirconia restorations treated with Biomic LiSi Connect. The cumulative survival rate was expressed as the percentage of restorations remaining clinically functional at the end of the three-year observation period.

In addition to overall survival, complications were analyzed descriptively by restorative material, anatomical location, preparation-design characteristics, and patient-reported satisfaction outcomes. This approach was intended to identify clinically relevant trends in restoration longevity, retention performance, and patient-perceived treatment outcomes.

Because survival included restorations that required recementation yet remained functional, survival outcomes were interpreted separately from complication-free success.

Because of the retrospective design and the limited sample size, survival outcomes were interpreted primarily descriptively and were intended to identify clinically relevant trends rather than to establish definitive causal relationships.

## 3. Results

### 3.1. Demographic Characteristics

Eleven patients completed the three-year evaluation: four received the lithium disilicate protocol (*N* = 4; *n* = 50 restorations) and seven received the zirconia protocol (*N* = 7; *n* = 50 restorations). No patient received both protocols. The cohort included seven female patients (63.6%) and four male patients (36.4%), with a mean age of 42.4 ± 7.1 years (range: 32–54 years).

A total of 100 ceramic veneers and partial-coverage restorations were included in the restoration-level analysis: 50 lithium disilicate restorations and 50 zirconia restorations treated with Biomic LiSi Connect.

Of the restorations included, 58 were maxillary and 42 mandibular. The lithium disilicate group comprised 33 anterior and 17 posterior restorations; the zirconia group comprised 37 anterior and 13 posterior restorations.

Anterior restorations predominated in both groups, but the anatomical distributions were not identical (Table 5). This imbalance, together with indication-based protocol allocation, precluded attribution of observed differences to the ceramic material alone.

### 3.2. Clinical Outcomes

Clinical complications recorded during follow-up are summarized as restoration-level counts, percentages, and descriptive Wilson 95% confidence intervals in Table 6 and Figure 5.

No fractures, chipping events, repairs, or replacements were observed in either group during the three-year period. This zero-event observation should not be interpreted as proof of absence of structural risk.

Marginal discoloration was the most frequently recorded finding, occurring in 17 lithium disilicate restorations (34%) and 19 zirconia restorations (38%). The observed frequencies were similar between groups.

Secondary caries was recorded in two lithium disilicate restorations (4%) and three zirconia restorations (6%). Hypersensitivity was observed in three lithium disilicate restorations (6%) and nine zirconia restorations (18%).

Debonding was the most clinically relevant retention-related complication. Six debonding events were recorded in the zirconia group (12%), whereas none occurred among lithium disilicate restorations (0%), corresponding to an absolute observed difference of 12 percentage points between protocols.

Overall, both restorative systems showed favorable three-year clinical performance. The principal difference between groups was related to retention, with debonding observed only among zirconia restorations treated with Biomic LiSi Connect.

### 3.3. Debonding Analysis

Because debonding was the only complication that clearly differed between the two restorative systems, a dedicated descriptive subanalysis was performed. Since all six debonding events occurred in zirconia restorations treated with Biomic LiSi Connect, this analysis was restricted to the zirconia group.

The anatomical distribution of debonding events is presented in Table 7.

Five of the six debonding events (83.3%) occurred in anterior restorations, and one event (16.7%) occurred in a posterior restoration. No debonding was recorded in the lithium disilicate group.

The restoration-level debonding incidence in the zirconia group was therefore 12% (6/50). All affected restorations were structurally intact, successfully recemented, and remained functional without replacement during the observation period.

The anatomical distribution of debonding events is illustrated in Figure 6, while a representative clinical example of zirconia restoration debonding and subsequent recementation is presented in Figure 7.

A representative debonding event observed in the zirconia group is illustrated in Figure 7.

The principal stages of laboratory and clinical reconditioning performed before recementation are illustrated in Figure 8.

### 3.4. Influence of Preparation Design on Retention

To explore factors potentially associated with loss of retention, zirconia restorations were analyzed according to preparation design. Because no debonding occurred in the lithium disilicate group, this subanalysis was limited to zirconia veneers and partial-coverage restorations treated with Biomic LiSi Connect.

For anterior restorations, three preparation designs (Type I–III) were evaluated according to the extent of proximal extension and the management of proximal contact areas. Posterior restorations were analyzed separately according to the degree of occlusal coverage. The results are summarized in Table 8 and Table 9.

Among the 37 anterior zirconia restorations, three debondings occurred in Type I preparations (3/15; 20.0%), two in Type II preparations (2/9; 22.2%), and none in Type III preparations (0/13). Among the 13 posterior zirconia restorations, no event occurred in the six Type I preparations, one occurred among six partial occlusal coverage restorations (1/6; 16.7%), and none occurred in the single complete-coverage restoration.

The observed events were distributed across preparation categories; however, the small number of events and unequal subgroup sizes precluded any inference regarding a progressive relationship between preparation extension and retention.

Among the 13 posterior zirconia restorations, no debonding occurred in the six Type I preparations, one event occurred in the partial occlusal coverage category (1/6; 16.7%), and the single restoration with complete occlusal coverage remained retained. The small and uneven subgroups did not permit meaningful comparison among posterior preparation designs.

Overall, preparation-design findings should be interpreted as exploratory. The limited number of debonding events, unequal category sizes, and clustering of multiple restorations within individual patients prevent causal interpretation.

A schematic summary of debonding rates according to preparation design is shown in Figure 9.

### 3.5. Survival Analysis

Restoration survival was assessed according to the predefined criterion of remaining in situ and in clinical function without replacement at the three-year follow-up (Table 10).

Although six debonding events occurred in the zirconia group, all affected restorations were successfully recemented and remained clinically functional throughout the follow-up period. Accordingly, these events were classified as complications rather than restoration failures, in accordance with the predefined outcome criteria.

Both lithium disilicate restorations and zirconia restorations treated with Biomic LiSi Connect achieved 100% three-year survival. However, survival should be interpreted separately from complication-free success because six zirconia restorations required recementation.

Representative clinical outcomes observed after three years of follow-up are illustrated in Figure 10 and Figure 11. The presented cases demonstrate satisfactory esthetic integration and maintenance of restoration integrity in both restorative systems.

### 3.6. Patient-Reported Outcomes

Patient-reported outcomes were collected once at the final three-year recall and are summarized at the patient level in Table 11 (lithium disilicate: *N* = 4; zirconia protocol: *N* = 7).

Patient-level scores were high in both groups. Because the questionnaire was study-specific, the sample was small, and no validated minimum clinically important difference was available, the results were not interpreted as evidence of equivalent patient-perceived performance.

The highest mean scores were observed for restoration integration within the smile and chewing comfort. Gingival appearance and tooth shape/proportions showed slightly lower values but remained highly rated. No clinically meaningful difference in final patient-reported satisfaction was evident between the two restorative systems.

Despite the higher frequency of debonding in the zirconia group, final patient satisfaction remained high after successful recementation of affected restorations (Figure 12).

## 4. Discussion

### 4.1. Overall Clinical Performance

This retrospective study compared the observed three-year outcomes of two complete adhesive restorative protocols. All 100 restorations remained in situ and functional, corresponding to 100% survival without replacement in each protocol. No replacement was required, but this outcome should not be interpreted as complication-free success.

The first null hypothesis was not supported descriptively for retention-related outcomes because six debondings occurred in the zirconia protocol and none in the lithium disilicate protocol. No difference was observed in survival without replacement or recorded structural failure. The second null hypothesis could not be formally tested because preparation subgroups were small and uneven, events were few, and restorations were clustered within patients.

No fractures, chipping events, or clinically apparent wear-related failures were recorded in either group. These findings indicate favorable structural performance for both ceramic systems during the three-year follow-up. They are consistent with previous clinical studies and systematic reviews reporting high survival rates and relatively low frequencies of catastrophic mechanical failure for adhesively bonded ceramic veneers and posterior partial-coverage restorations [2,3,4,5,6,7]. However, the present follow-up period should be interpreted as medium-term rather than long-term.

The absence of fracture in both groups is notable given the different ceramic compositions. Lithium disilicate is an etchable glass ceramic with an established adhesive protocol, whereas zirconia is a polycrystalline ceramic with high intrinsic strength but more challenging adhesive behavior [8,10,11,12,13,14]. Under the clinical conditions of this study, both materials appeared to provide sufficient structural resistance during the three-year observation period.

Nevertheless, identical survival rates should not be interpreted as identical clinical behavior. Six zirconia restorations debonded, whereas no debonding was observed among lithium disilicate restorations. Because all debonded zirconia restorations were successfully recemented and remained in function, these events were classified as complications rather than failures according to the predefined survival criteria.

The principal observed differences concerning retention rather than ceramic integrity. This finding compares two clinically selected protocols, including different materials, conditioning steps, resin cements, indications, and anatomical distributions, and cannot be interpreted as an isolated causal effect of zirconia or Biomic LiSi Connect.

### 4.2. Mechanical Reliability of Both Veneer Systems

During the three-year follow-up, no fractures, chipping events, wear-related degradation, repairs, or replacements were recorded in either study group. Both lithium disilicate and zirconia restorations therefore maintained their structural integrity throughout the observation period. This finding is consistent with previous clinical studies and systematic reviews reporting high survival rates and low rates of catastrophic mechanical failure for ceramic veneers and posterior partial-coverage restorations [2,3,4,5,6,7,18,19,20].

The absence of mechanical complications in the lithium disilicate group agrees with the established clinical performance of this material in anterior and posterior indications. Layton and Clarke reported estimated five-year survival rates above 90% for non-feldspathic ceramic veneers, while emphasizing substantial heterogeneity among studies in patient selection, preparation design, bonding substrate, and failure criteria [18]. Similarly, Fabbri et al. reported high survival and success rates for lithium disilicate veneers, onlays, and crowns in a retrospective evaluation of 860 anterior and posterior restorations [19]. Although fracture and chipping remain recognized failure mechanisms, they are relatively uncommon when material selection, preparation design, and adhesive cementation are appropriately controlled.

The zirconia restorations also showed no fracture or chipping during follow-up. Although zirconia has greater flexural strength and fracture toughness than lithium disilicate, this mechanical advantage did not translate into a measurable clinical difference in structural outcomes because neither group experienced structural failure. Therefore, the present results should not be interpreted as evidence of mechanical equivalence between the two ceramics, but rather as evidence that both materials provided adequate short- to medium-term strength under the specific clinical conditions evaluated.

A possible explanation for the favorable structural outcomes is the reinforcing effect of adhesive bonding. When thin ceramic restorations are bonded effectively, functional loads are distributed across the ceramic, resin cement, and tooth structure rather than being borne by the ceramic alone [8,9]. Consequently, the mechanical performance of partial-coverage restorations depends on multiple interacting factors, including enamel preservation, preparation geometry, ceramic thickness, adhesive protocol, and occlusal loading. The absence of fractures in this cohort likely reflects the combined influence of these factors rather than the inherent strength of either material alone.

Structural failure therefore did not differentiate the two restorative systems during the first three years of clinical service. The clinically relevant distinction was loss of retention, which occurred only in the zirconia group while the ceramic restorations themselves remained intact.

### 4.3. Adhesive Performance of Zirconia Veneers and Partial-Coverage Restorations: Retention as the Critical Clinical Challenge

The main clinical difference between the two restorative systems was the occurrence of debonding exclusively in the zirconia group. During the three-year follow-up, six of fifty zirconia restorations treated with Biomic LiSi Connect debonded, corresponding to a restoration-level incidence of 12%. No debonding was recorded among lithium disilicate restorations. All six affected zirconia restorations were structurally intact, were successfully recemented, and remained functional at the final evaluation. Thus, debonding affected complication-free performance but did not reduce the predefined restoration survival rate.

This finding emphasizes the distinction between ceramic strength and adhesive reliability. Zirconia has greater intrinsic strength and fracture toughness than lithium disilicate, but these properties do not necessarily improve retention. For adhesively bonded partial-coverage restorations, clinical stability depends on both the resin cement-tooth interface and the resin cement-restoration interface. Failure at either interface may result in debonding without structural damage to the ceramic restoration.

Unlike lithium disilicate, zirconia lacks a silica-containing glass phase and is therefore not predictably conditioned using the conventional hydrofluoric acid-silane protocol. Bonding to zirconia usually requires surface pretreatment. Airborne particle abrasion can increase surface roughness and available bonding area, while phosphate-containing functional monomers such as 10-MDP can interact chemically with zirconium oxide [11,12,13,14,21]. In vitro studies generally indicate that combined micromechanical and chemical conditioning improves bond strength more effectively than either approach alone; however, outcomes depend on zirconia composition, air-abrasion parameters, resin cement, testing method, and artificial aging protocol [13,14,21].

Glass-ceramic surface coating represents another strategy for improving zirconia bonding. Instead of relying solely on direct chemical interaction with zirconia, a thin glass-ceramic layer is applied to the intaglio surface to create an etchable substrate that can interact with silane and resin cement. Experimental studies have reported improved bond strength and aging behavior for coated zirconia compared with uncoated specimens [13,21]. This provides a rationale for the Biomic LiSi Connect approach used in the present study. Nevertheless, in vitro bond-strength values cannot fully reproduce the combined effects of moisture, thermal variation, cyclic loading, substrate variability, restoration geometry, and operator-dependent factors encountered clinically [16].

The observed debonding events should not be interpreted as evidence that the surface-modification system was ineffective. The coating may have contributed to the retention of most zirconia restorations; however, the study did not include an untreated zirconia control group and therefore could not isolate the specific effect of Biomic LiSi Connect. In addition, the two restorative groups differed in ceramic composition, surface treatment, resin cement, clinical indication, substrate characteristics, and preparation design.

Artificial aging studies have shown that resin-zirconia bond strength can decrease after thermocycling or prolonged water exposure despite favorable initial results [21,22,23]. This may help explain why debonding can persist as a clinical complication even when surface conditioning is performed.

Other potential contributors include contamination during try-in, incomplete surface cleaning, variation in coating thickness or conditioning, limited enamel availability, and functional loading. These explanations remain speculative in the present study because the exact debonding interface was not examined microscopically or chemically.

The successful recementation of all affected restorations indicates that the complication was clinically manageable and did not necessitate restoration replacement. However, recementation still represents an unplanned intervention and should not be regarded as equivalent to complication-free service. Within the limits of this retrospective cohort, zirconia restorations treated with Biomic LiSi Connect achieved complete three-year survival and remained structurally stable but showed a higher observed frequency of retention-related complications than lithium disilicate restorations.

Further prospective studies using standardized preparation designs, identical luting conditions, patient-level adjustment, and detailed failure-interface analysis are required to determine the relative contributions of the ceramic, coating, cementation protocol, and preparation geometry.

### 4.4. Influence of Preparation Design on Veneer and Partial-Coverage Restoration Retention

The distribution of debonding events was explored according to preparation geometry and region. Among the 37 anterior zirconia restorations, three events occurred in Type I preparations (3/15; 20.0%), two in Type II preparations (2/9; 22.2%), and none in Type III preparations (0/13). Among the 13 posterior restorations, no event occurred in the six Type I preparations, one occurred in the partial occlusal coverage category (1/6; 16.7%), and the single complete-coverage restoration remained retained.

These small, uneven subgroups do not support a progressive relationship between preparation extension and retention. Preparation extension was used only as a surrogate for potential bonding area, which was not measured directly.

Moreover, only six events were available for analysis, the preparation categories were unequally represented, and multiple restorations originated from the same patients. Consequently, the study was not adequately powered to establish a statistically reliable association between preparation design and debonding.

Nevertheless, preparation geometry has a plausible biomechanical role in the performance of adhesively retained restorations. Changes in proximal, incisal, and occlusal extension modify the available bonding area, the proportion of enamel and dentin exposed, the path of insertion, ceramic thickness, and the distribution of functional stresses across the restoration-cement-tooth complex. Laboratory investigations have shown that preparation design and the condition of the remaining tooth structure can influence the load-bearing behavior of ceramic veneers [24]. However, greater extension does not necessarily produce a more favorable mechanical response in every situation, because the benefits of an increased bonding area must be balanced against the removal of sound enamel and the creation of geometrical regions that may concentrate stress [9,24].

The same principle applies to posterior partial-coverage restorations. A systematic review of ceramic occlusal veneers found that preparation design can affect marginal adaptation and fracture resistance, although substantial heterogeneity among laboratory protocols prevented identification of a single superior configuration [25]. The available evidence is predominantly in vitro and focuses more often on fracture behavior than on clinical debonding. Therefore, it does not directly establish that complete occlusal coverage improves the retention of zirconia restorations in vivo.

The observed pattern may reflect substrate quality, enamel distribution, tooth position, occlusal contacts, ceramic thickness, and indication-based selection. Because bonding area was not measured directly and preparation designs were not randomized, preparation extension should be regarded only as a surrogate for potential bonding area.

The findings nevertheless identify a clinically relevant question. Existing literature indicates that veneer performance is influenced by the interaction among preparation design, ceramic material, and the substrate available for bonding rather than by any of these factors in isolation [9,24,25,26]. This interaction may be especially important for zirconia, for which adhesive conditioning remains more technique-sensitive than for silica-based ceramics. The present results suggest that preparation design deserves consideration in future clinical investigations of zirconia partial-coverage restorations, but they do not justify intentional extension of the preparation solely to increase retention.

From a clinical perspective, enamel preservation should remain the primary objective. Preparation extension should be determined by restorative space, substrate condition, esthetic requirements, management of proximal contacts, and occlusal demands rather than by a general assumption that a larger preparation will necessarily provide better retention. Prospective studies with balanced preparation groups, standardized occlusal assessment, patient-level statistical adjustment, and a larger number of events are required to determine whether the pattern observed in this cohort represents a reproducible clinical effect.

### 4.5. Clinical Implications and Patient-Reported Outcomes

The results reinforce the importance of evaluating restorative systems as integrated clinical protocols rather than selecting materials solely on the basis of strength. Although three-year survival was identical for lithium disilicate and zirconia restorations, their complication profiles differed. Lithium disilicate restorations showed no debonding in this cohort, whereas six zirconia restorations required recementation. Material selection should therefore consider bonding substrate, optical demands, preparation design, surface-conditioning protocol, cementation technique, and expected functional loading.

Lithium disilicate appeared more predictable from an adhesive perspective in the present study. Its established hydrofluoric acid-silane conditioning protocol and the availability of enamel for bonding may explain the absence of debonding [8,9]. These findings support the use of lithium disilicate for anterior and posterior partial-coverage restorations when substrate color can be adequately managed and predominantly enamel-bonded preparations are feasible. However, the absence of complications during a three-year period does not exclude the possibility of longer-term failures due to fracture, marginal degradation, or debonding [3,4,5,6,7,18,19,20].

Zirconia restorations treated with Biomic LiSi Connect maintained structural integrity and function in all cases, including those requiring recementation. These results suggest that this approach may be clinically useful when the masking capacity or mechanical properties of zirconia are required. However, the 12% restoration-level incidence of debonding highlights the continued clinical importance of retention.

The present study does not demonstrate equivalence between Biomic LiSi Connect-treated zirconia and lithium disilicate, nor does it establish zirconia as the preferred material for partial-coverage restorations. Instead, it underscores the need for careful case selection and strict adherence to the complete adhesive protocol.

Both groups showed high patient-reported satisfaction. Mean VAS scores ranged from 9.3 to 9.8 across domains including overall satisfaction, smile appearance, tooth color and shape, natural appearance, chewing comfort, speech, gingival appearance, and willingness to repeat or recommend treatment. Visual Analog Scales are widely used in prosthodontics because they are simple to administer and can capture patient perceptions [17]. However, VAS scores may be influenced by expectations, response style, recall bias, and clustering of responses near the upper end of the scale [27].

The similarity of patient-reported scores suggests high final acceptability of both restorative approaches in the selected patients. However, these scores do not prove that debonding had no effect on patient experience. The questionnaire was administered during follow-up rather than before or immediately after complications occurred, and all debonded restorations had been successfully recemented by the time outcomes were assessed. Therefore, the scores reflect the subjective evaluation of the final clinical condition rather than the immediate inconvenience, concern, or additional intervention associated with debonding.

Interpretation of the patient-reported outcomes is further limited by the small sample size and the use of a non-validated, study-specific questionnaire. The very high scores and low standard deviations may also indicate a ceiling effect. Consequently, the PROM findings should be interpreted descriptively and should not be considered proof of equivalent patient-perceived performance between restorative systems.

From a practical perspective, both materials produced favorable esthetic and functional outcomes at three years, but through restorative protocols with different adhesive behavior. Lithium disilicate offered more consistent retention in this cohort, while zirconia provided structural stability and satisfactory final patient perception despite requiring recementation in six cases. Treatment planning should therefore balance material properties, adhesive predictability, substrate characteristics, and patient expectations rather than prioritizing any single factor.

### 4.6. Strengths, Limitations, and Future Perspectives

This study has several strengths. It addresses a clinically relevant question for which medium-term evidence remains limited: the performance of zirconia veneers and partial-coverage restorations after lithium silicate surface modification. The three-year observation period allowed assessment of mechanical complications, retention-related events, survival, and patient-reported outcomes. In addition, the exploratory analysis of debonding according to preparation design generated clinically relevant hypotheses regarding the relationship between restoration geometry and retention.

The study also has important limitations.

The cohort was a non-consecutive convenience sample. Fifty zirconia restorations were selected from a larger pool to provide an equal restoration count and anatomical representation, introducing selection bias. Exact zirconia composition, yttria content, thickness, batch numbers, detailed firing records, and complete air-abrasion parameters were unavailable. Enamel/dentin proportions, existing composites, endodontic status, substrate shade, bruxism/nightguard use, exact maintenance-visit counts, and event dates were not consistently recorded. These omissions limit control of confounding and prevent time-to-event analysis.

First, although 100 restorations were evaluated, they were clustered within only 11 patients, with four patients in the lithium disilicate group and seven in the zirconia group. Five of seven Zirconia patients experienced at least one debonding. The apparently large restoration count should not obscure the limited number of independent patient clusters.

Second, material selection was based on clinical indication rather than random allocation. Differences in substrate shade, remaining enamel, tooth position, preparation requirements, occlusal conditions, and aesthetic demands may therefore have influenced the results. Surface conditioning and luting protocols also differed between groups, meaning that the study compared two restorative systems rather than isolating the effect of material alone.

Third, no control group of untreated zirconia or zirconia treated with an alternative bonding protocol was included. The specific contribution of Biomic LiSi Connect to retention therefore cannot be determined. In addition, the debonding interfaces were not examined microscopically or chemically, so it was not possible to identify whether failure occurred at the zirconia-cement interface, within the cement layer, or at the tooth-cement interface.

Fourth, the preparation-design categories were unevenly represented. Only seven anterior restorations were classified as Type III, and only one posterior restoration had complete occlusal coverage. With only six debonding events available for analysis, the study was not sufficiently powered to establish an association between preparation design and retention. The observed pattern should therefore be considered hypothesis-generating rather than causal.

Fifth, clinical outcomes were assessed retrospectively rather than through a prospective, blinded, and calibrated evaluation protocol. The customized VAS questionnaire was not psychometrically validated and may have been affected by a ceiling effect. Moreover, patient-reported outcomes were recorded after clinical management of complications and therefore do not capture the immediate impact of debonding or recementation.

The definition of survival also requires careful interpretation. Restorations that were recemented but remained functional and did not require replacement were counted as surviving. Accordingly, the 100% survival rate should not be interpreted as a 100% complication-free success rate. Future studies should report survival and intervention-free success separately to provide a more complete description of clinical performance.

Finally, follow-up was restricted to the documented three-year recall. Exact event dates and time to debonding were not collected in the analytic dataset, so Kaplan–Meier or mean time-to-event analyses could not be performed. The single-practice setting and selected cohort limit external validity.

Future investigations should include multicenter prospective designs, larger patient populations, balanced preparation groups, split-mouth or otherwise appropriately con-trolled designs where clinically and ethically feasible, identical or factorially controlled luting conditions, standardized FDI or modified USPHS evaluation criteria, and cluster-aware statistical methods that account for multiple restorations within patients. Comparisons among untreated zirconia, 10-MDP-based protocols, and lithium silicate sur-face-modification systems would help clarify the role of individual bonding strategies.

Failure-interface analysis, longer observation periods, and validated patient-reported outcome measures would further improve the clinical relevance of future studies. Despite these limitations, the present study provides preliminary clinical evidence regarding zirconia partial-coverage restorations treated with Biomic LiSi Connect. The system was associated with complete three-year restoration survival and favorable final patient-reported outcomes, although retention-related complications occurred more frequently than with lithium disilicate partial-coverage restorations.

These findings support further investigation of adhesive durability and preparation geometry but should not be considered definitive evidence of equivalence or superiority between the two restorative systems.

## 5. Conclusions

In this selected retrospective convenience cohort, no replacements or structural failures were observed among 50 lithium disilicate and 50 zirconia restorations treated with Biomic LiSi Connect for three years. Both protocols therefore showed 100% survival without replacement (95% CI: 92.9–100.0%). However, six zirconia restorations required recementation after debonding, whereas no debonding was recorded among lithium disilicate restorations. Because restorations were clustered within 11 patients and the protocols differed in material, surface conditioning, cementation, clinical indication, and anatomical distribution, the observed findings are exploratory and should not be interpreted as an isolated causal material effect.

## Figures and Tables

**Figure 1:**
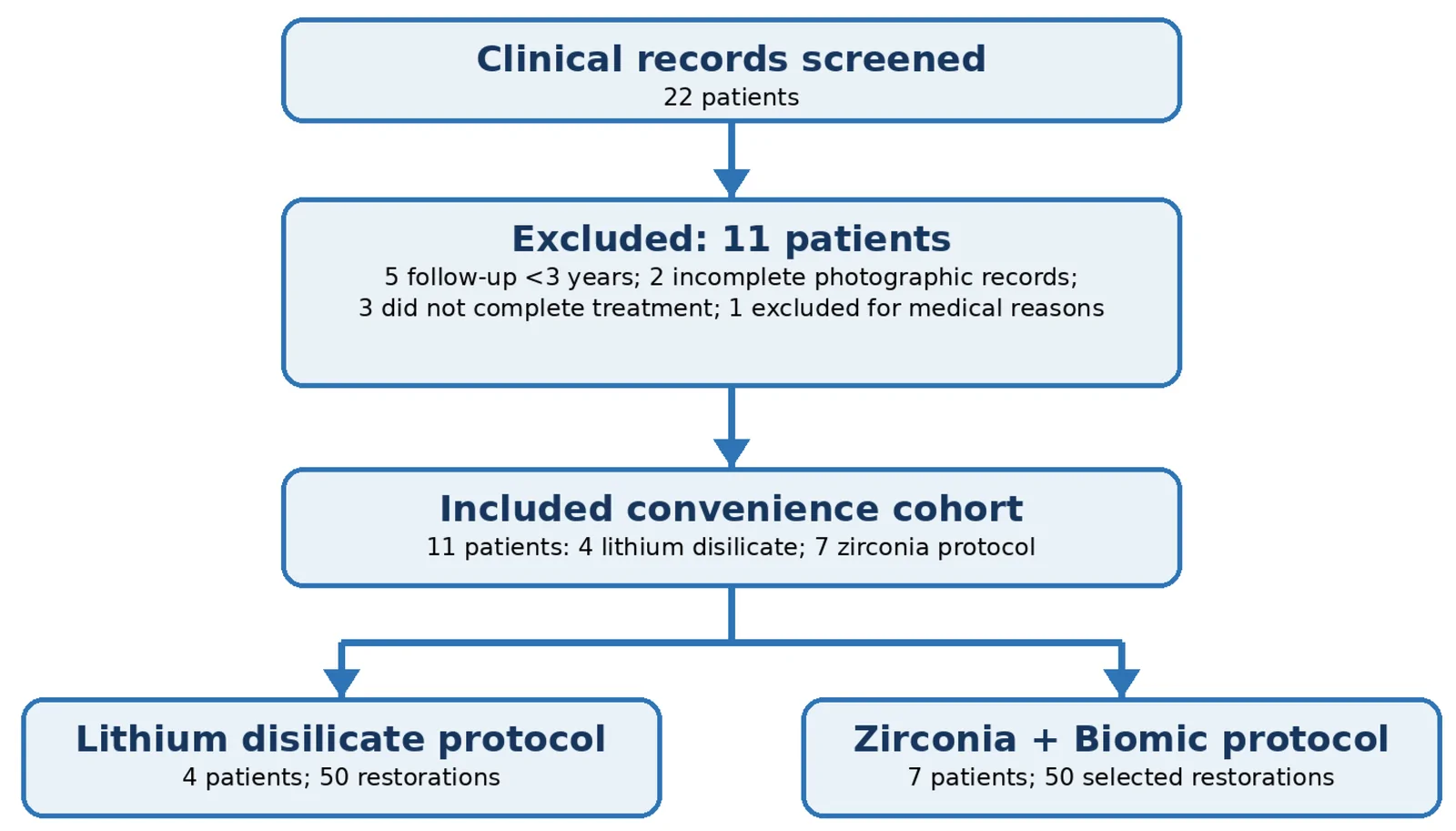
STROBE-style flow diagram of retrospective case selection and restoration sampling.

**Figure 2:**
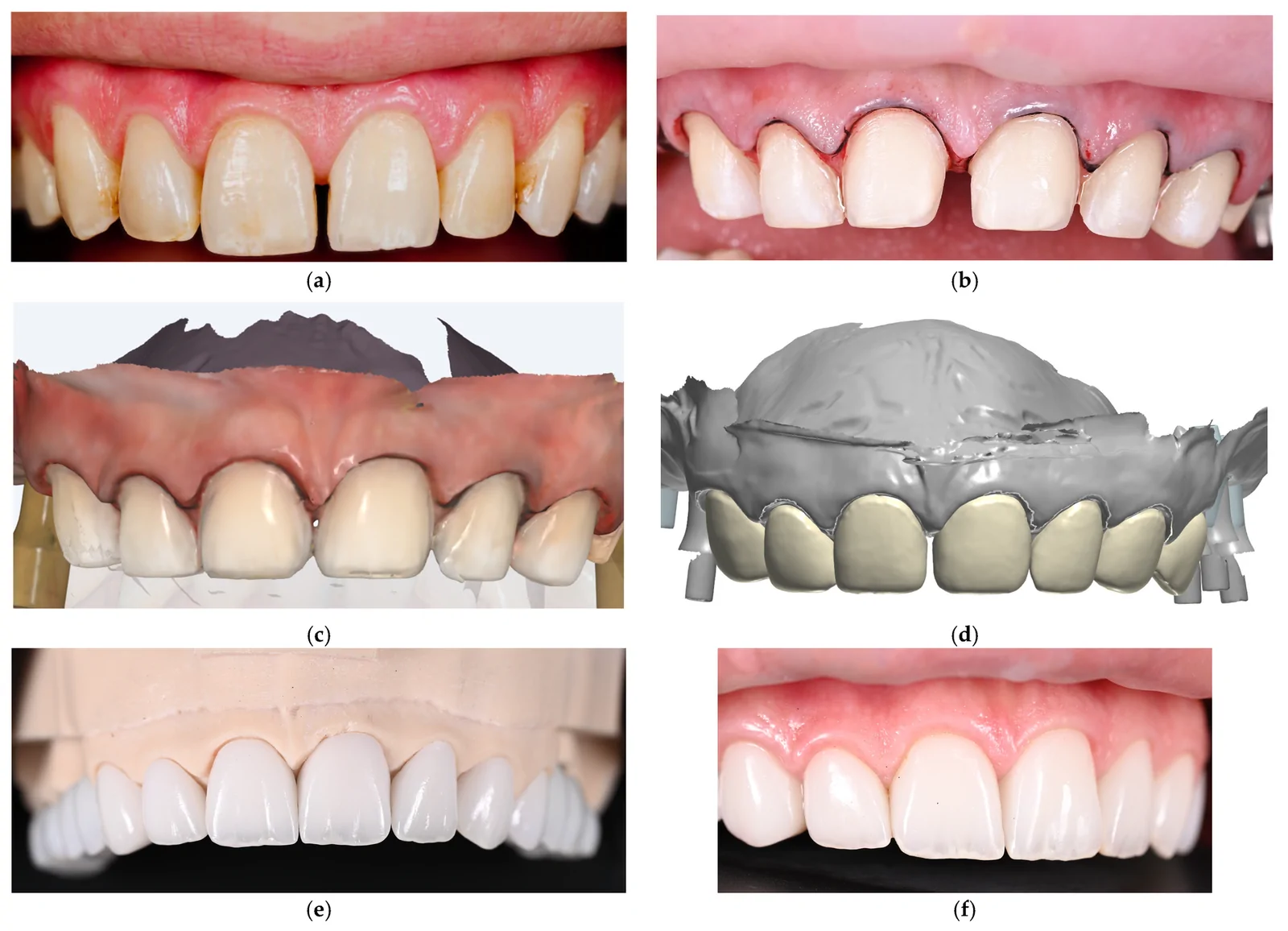
Digital treatment planning and mock-up-guided workflow: (**a**) initial clinical situation; (**b**) tooth preparation guided by the planned restoration design; (**c**) digital scan of the prepared teeth; (**d**) virtual mock-up and digital design of the definitive restorations; (**e**) restorations on the working model before cementation; and (**f**) final clinical outcome after adhesive cementation.

**Figure 3:**
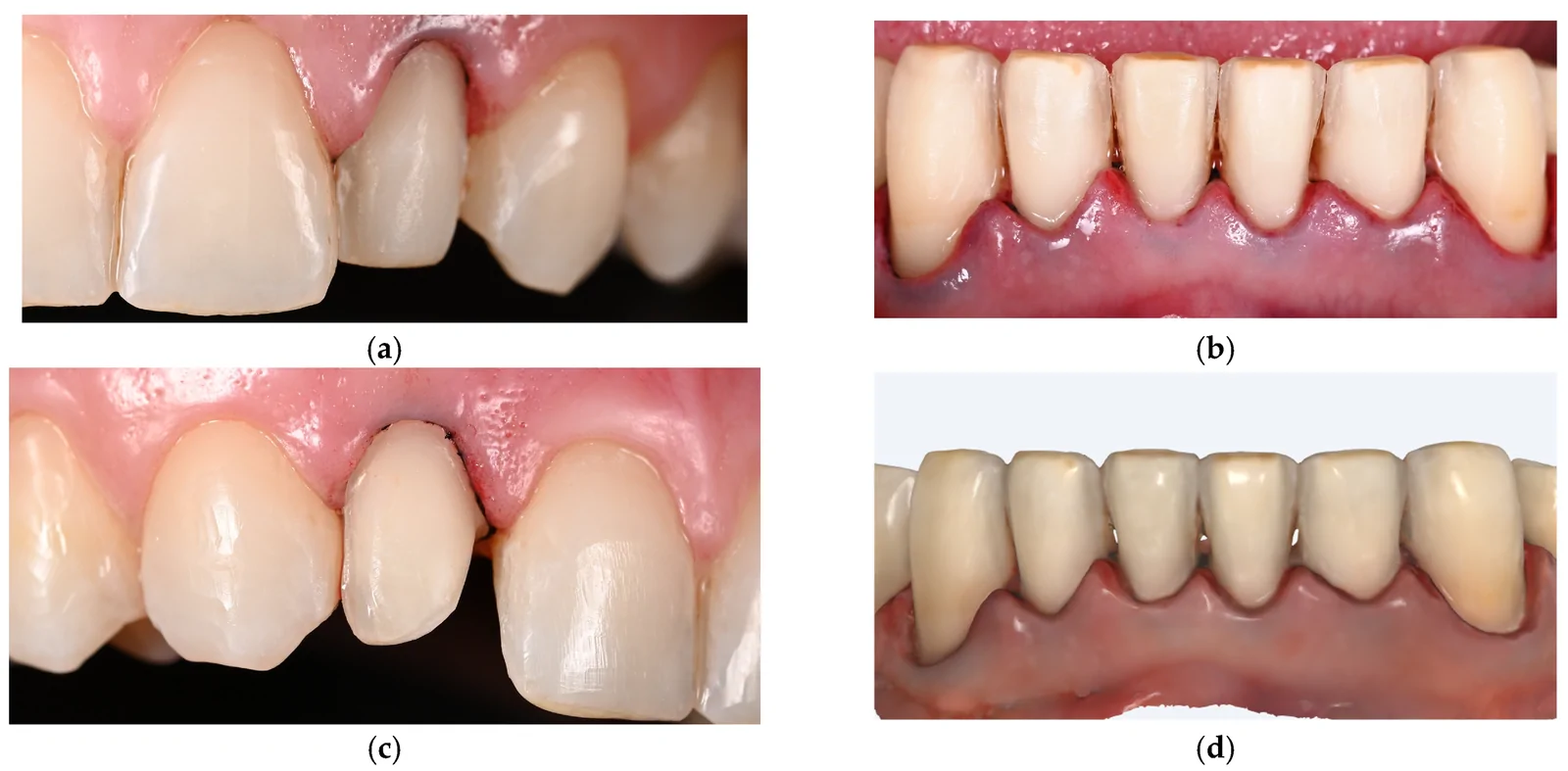
Representative preparation designs used for ceramic veneers and partial-coverage restorations: (**a**) Type I preparation with partial proximal extension and preservation of contact points; (**b**) Type II preparation with complete proximal extension to the palatal surface and preservation of contact points; (**c**) Type III preparation with complete proximal extension and elimination of contact points; and (**d**) digital scan of definitive preparations.

**Figure 4:**
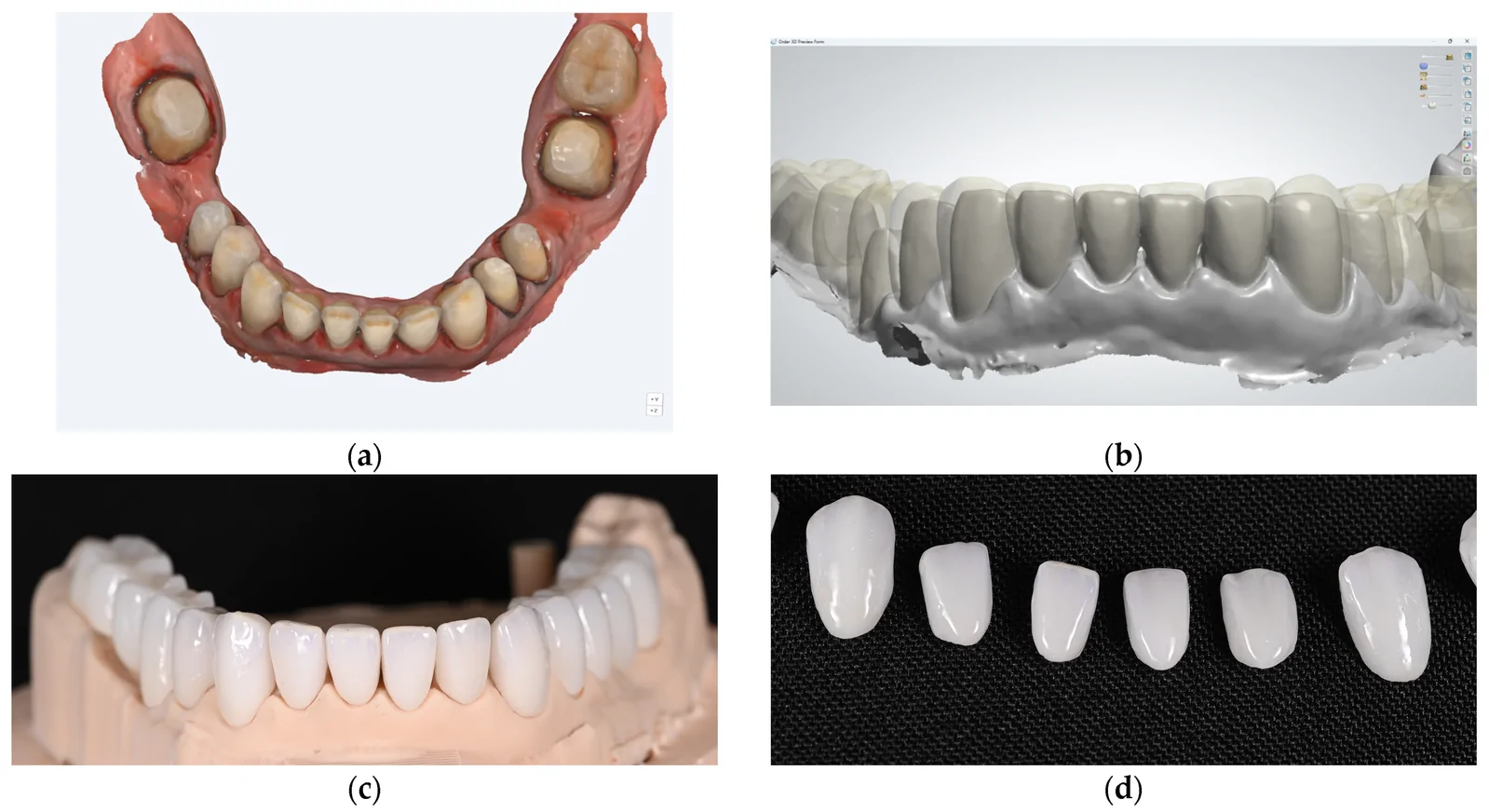
Representative digital planning and laboratory workflow for ceramic veneers and partial-coverage restorations: (**a**) digital scan after tooth preparation; (**b**) virtual design of the definitive restorations; (**c**) final restorations on the printed model; and (**d**) individual ceramic restorations before adhesive cementation.

**Figure 5:**
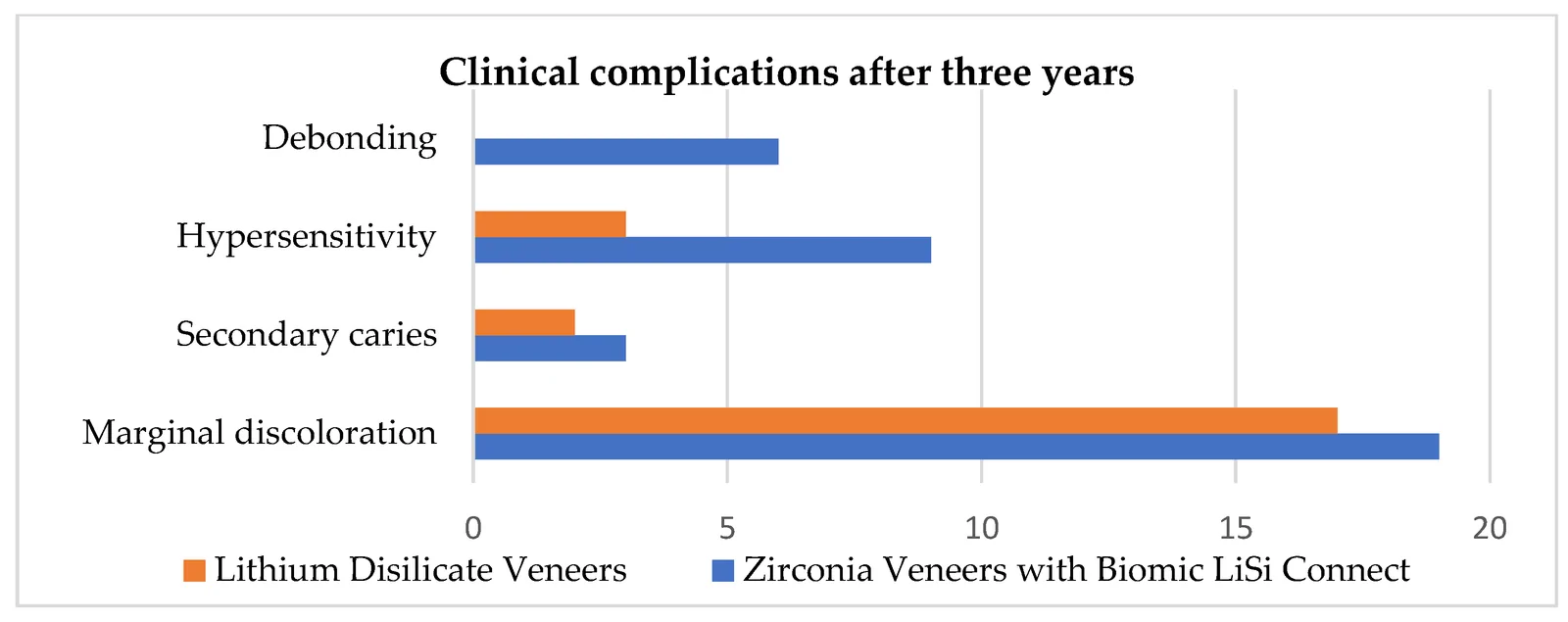
Comparison of clinical complications recorded in lithium disilicate and zirconia restorations after three years of follow-up.

**Figure 6:**
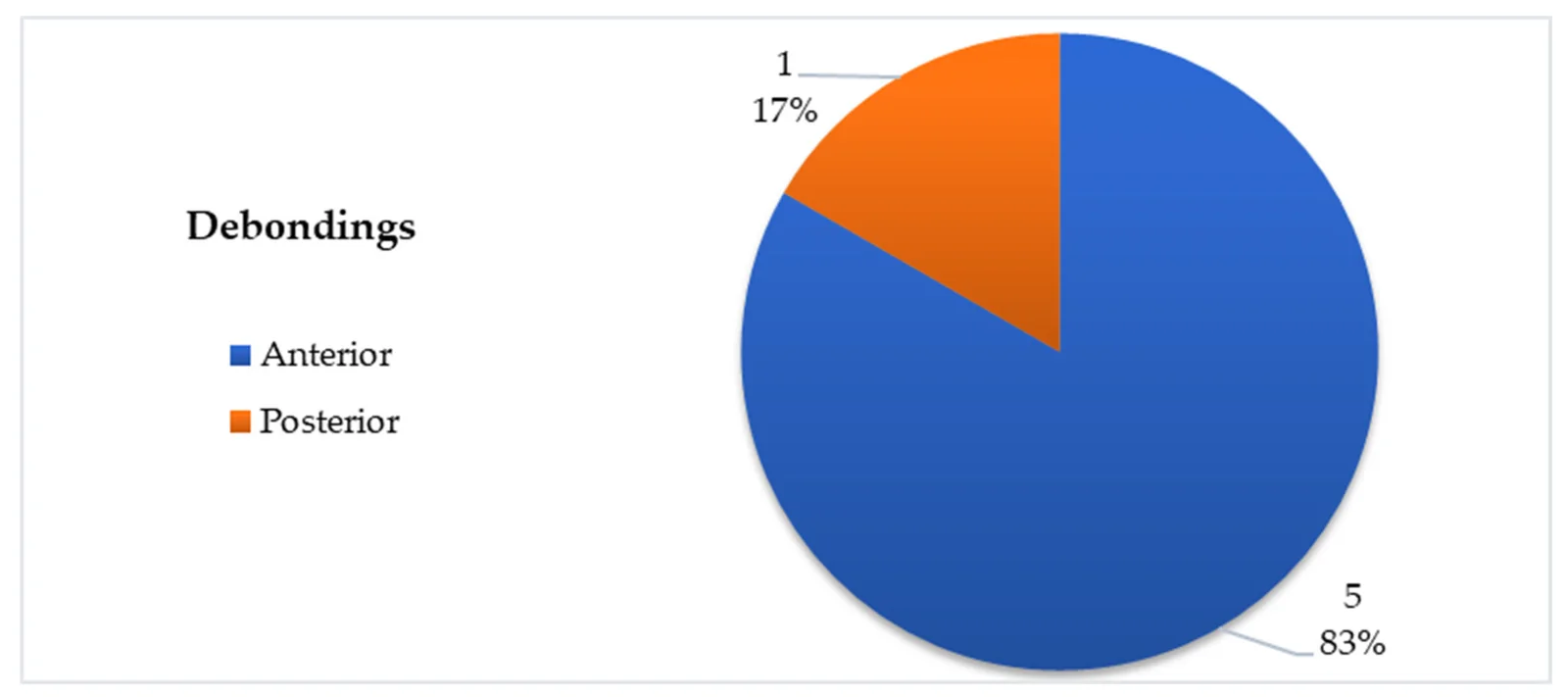
Anatomical distribution of debonding events observed in zirconia restorations treated with Biomic LiSi Connect after three years of follow-up.

**Figure 7:**
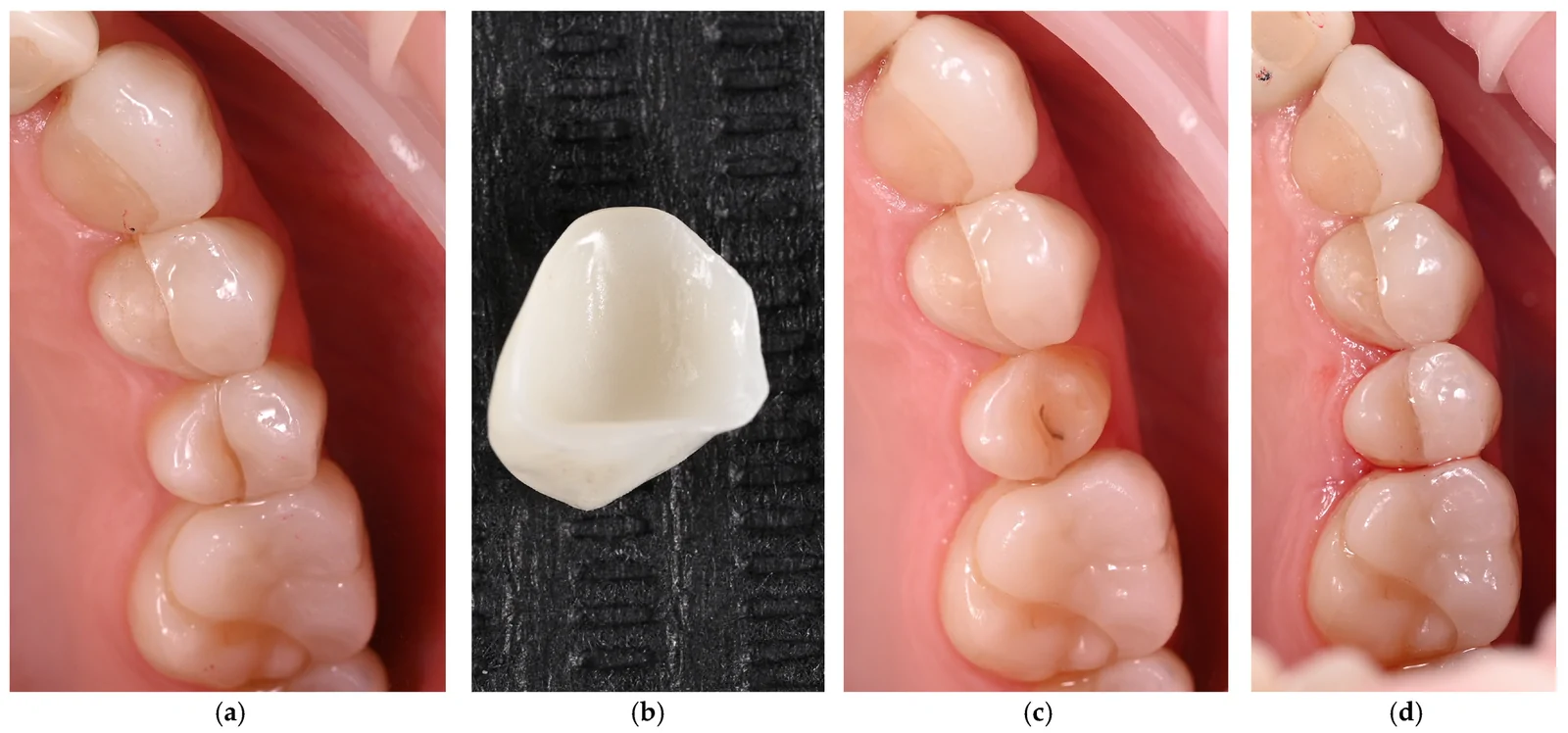
Clinical example of zirconia restoration debonding and management: (**a**) restoration in clinical function before debonding; (**b**) restoration after loss of retention; (**c**) clinical appearance after debonding; and (**d**) restoration after recementation.

**Figure 8:**
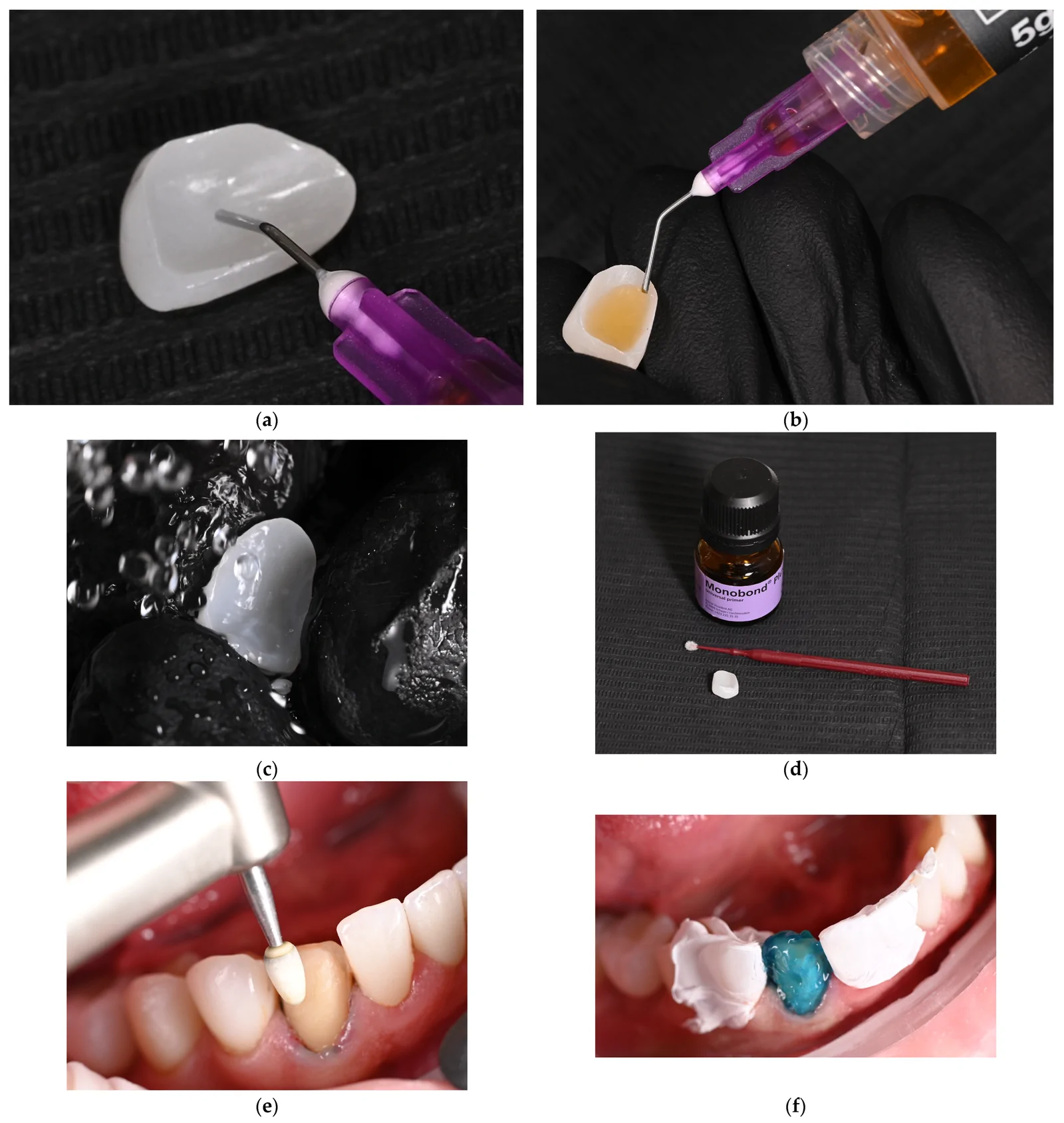

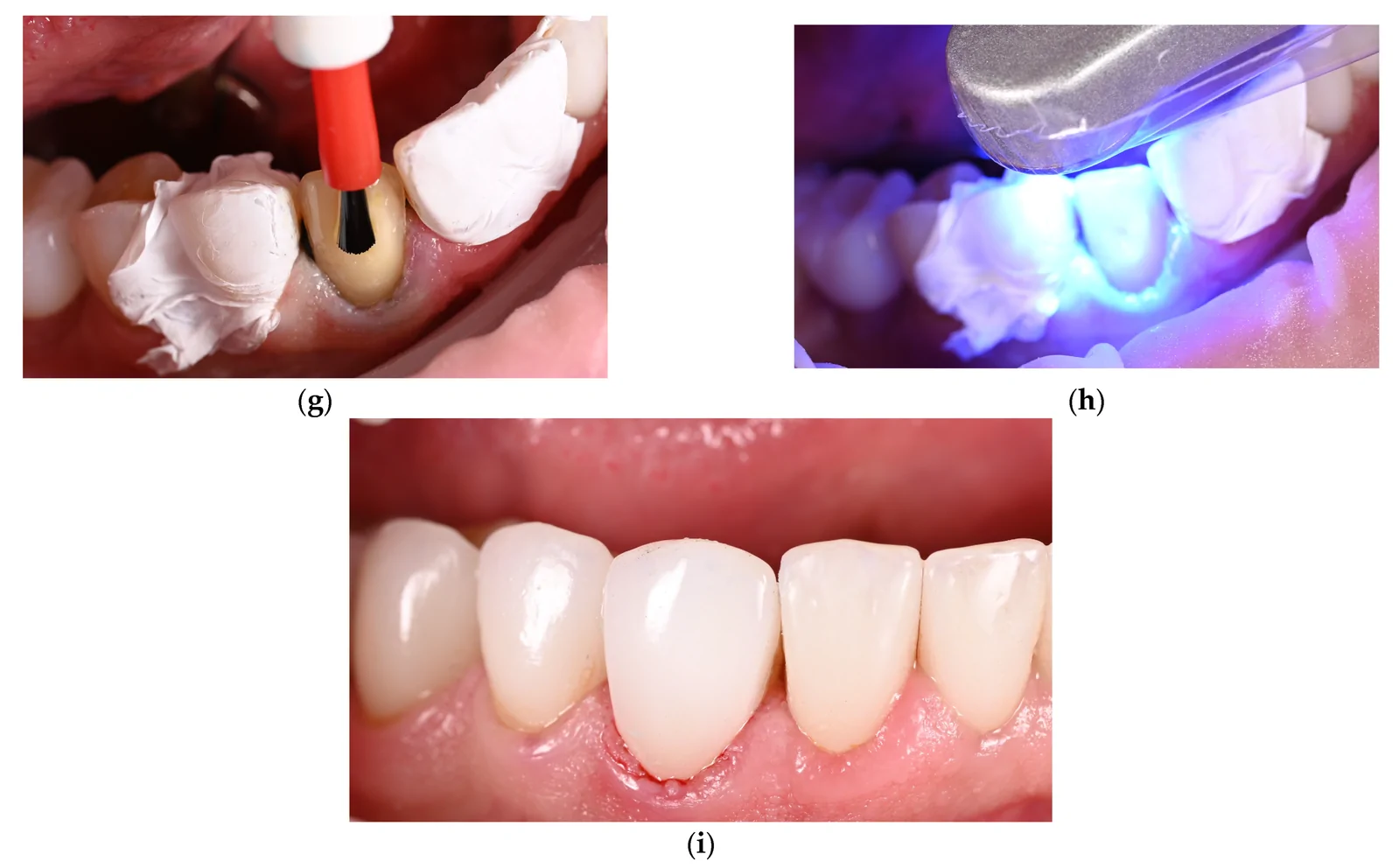
Representative stages of zirconia restoration reconditioning and recementation: (**a**) intaglio surface after ultrasonic decontamination, reapplication of Biomic LiSi Connect, and laboratory air abrasion; (**b**) application of 5% hydrofluoric acid; (**c**) rinsing and ultrasonic cleaning; (**d**) application of Monobond Plus; (**e**) refinement of the dental substrate using an Arkansas stone; (**f**) phosphoric acid etching of the dental surface; (**g**) application of Adhese Universal; (**h**) light polymerization; and (**i**) clinical appearance after recementation.

**Figure 9:**
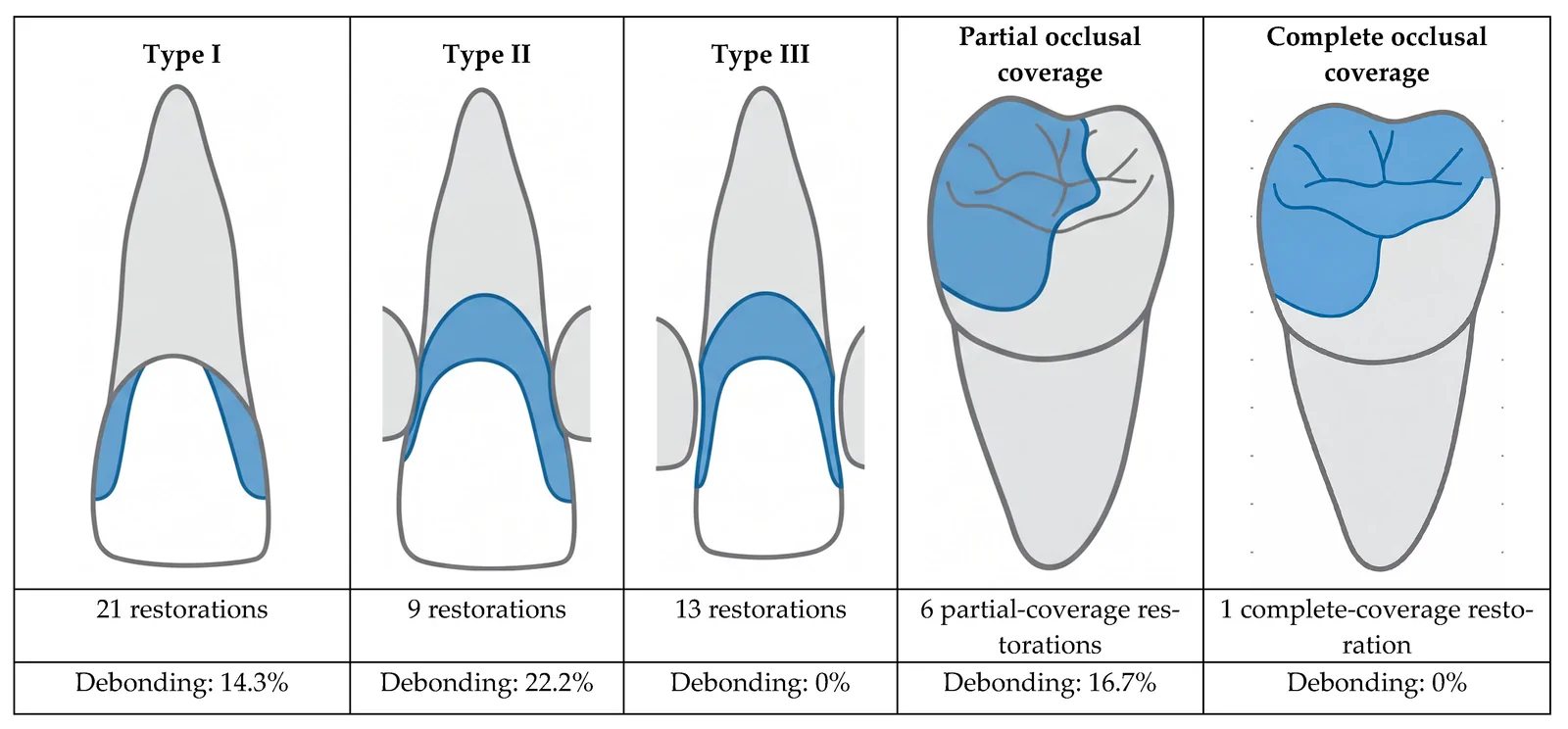
Debonding rates according to preparation-design category in all zirconia restorations. The Type I total includes 15 anterior and 6 posterior restorations; Type II and Type III were anterior, whereas partial and complete occlusal coverage categories were posterior.

**Figure 10:**
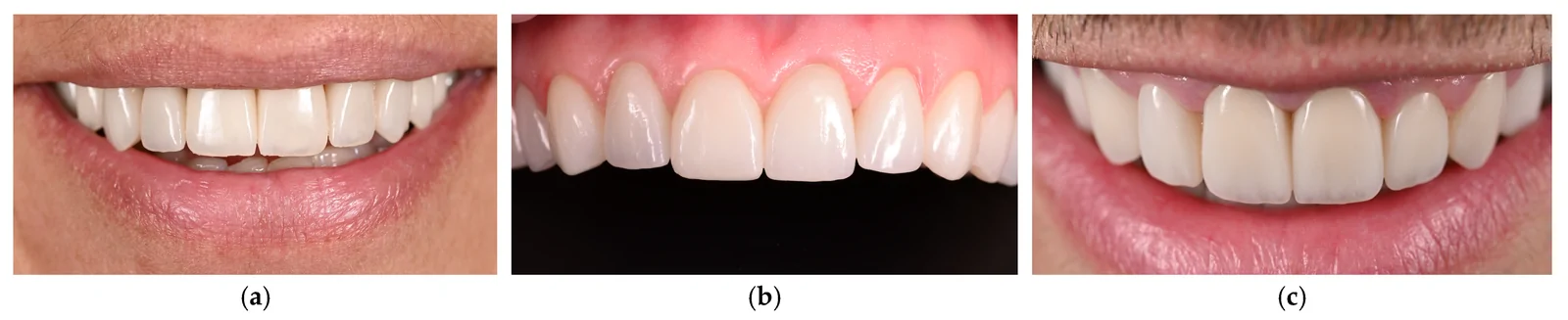
Representative clinical outcomes of lithium disilicate restorations after three years of follow-up: (**a**–**c**) examples showing favorable esthetic integration, marginal adaptation, and soft tissue response.

**Figure 11:**
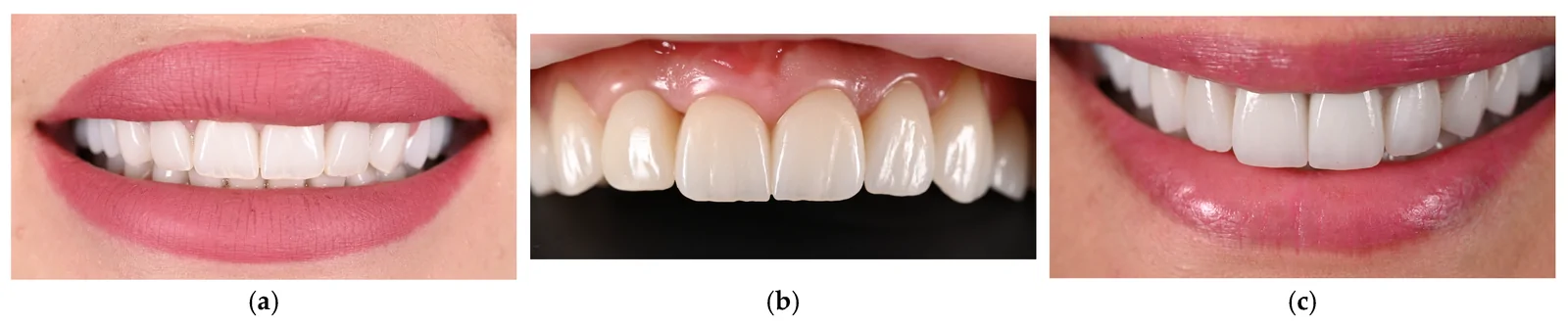
Representative clinical outcomes of zirconia restorations treated with Biomic LiSi Connect after three years of follow-up: (**a**–**c**) examples showing satisfactory esthetic and functional performance at recall.

**Figure 12:**
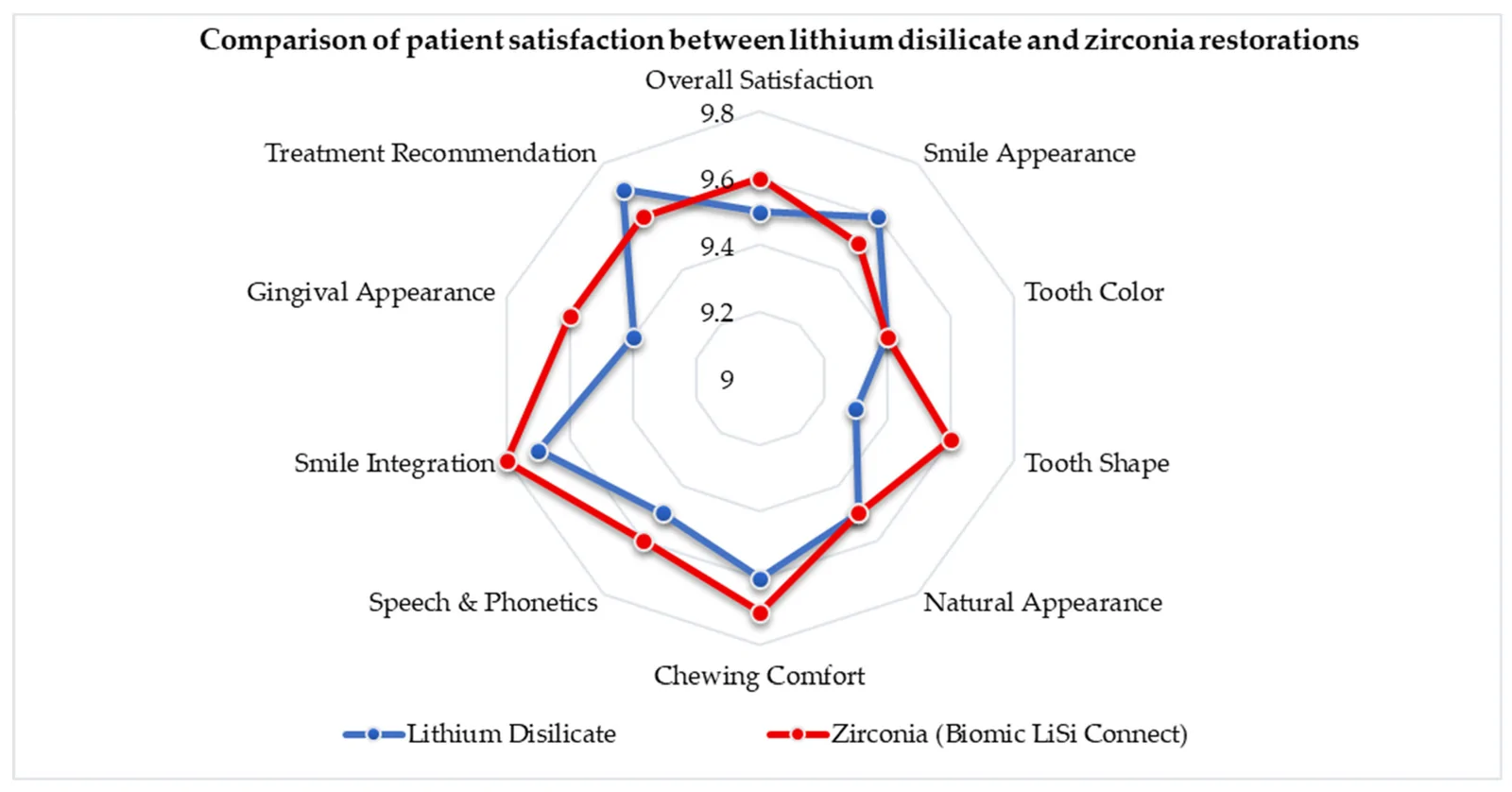
Radar plot comparing patient-reported outcome measures assessed using the Visual Analog Scale between lithium disilicate restorations and zirconia restorations treated with Biomic LiSi Connect after three years of follow-up.

**Table 1:** Demographic characteristics of the included patients.

Patient ID	Sex	Age (Years)	Restorative Material	Restorations, *n*	Debondings, *n*
P1	Female	32	Lithium disilicate	5	0
P2	Female	34	Lithium disilicate	13	0
P3	Male	36	Lithium disilicate	24	0
P4	Female	39	Lithium disilicate	8	0
P5	Female	41	Zirconia	8	0
P6	Male	42	Zirconia	3	1
P7	Female	42	Zirconia	7	2
P8	Female	46	Zirconia	6	0
P9	Male	48	Zirconia	6	1
P10	Female	52	Zirconia	7	1
P11	Male	54	Zirconia	13	1
Total/Mean	7 Female/4 Male	42.4 ± 7.1	11 patients	100	6

**Table 2:** Distribution of restorations according to dental arch and restorative material.

Dental Arch	Lithium Disilicate Restorations	Zirconia Restorations with Biomic LiSi Connect	Total
Maxillary	30	28	58
Mandibular	20	22	42
Total	50	50	100

**Table 3:** Definitions of clinical outcome measures used for restoration assessment.

Outcome Measure	Definition
Fracture	Structural failure involving the ceramic restoration that compromised its integrity or clinical function.
Chipping	Localized loss of ceramic material without complete fracture of the restoration.
Marginal Discoloration	Visible marginal staining recorded as present/absent at the three-year clinical examination and review of standardized photographs; no validated FDI/USPHS severity scale was applied.
Wear Surface Degradation	Clinically or photographically visible surface deterioration beyond the baseline appearance; physiological antagonist attrition was not quantified separately.
Secondary Caries	Clinical diagnosis of caries adjacent to a restoration margin, supported by radiographic information when available in the record.
Hypersensitivity	Patient-reported sensitivity to thermal, chemical, or mechanical stimuli present at the final three-year recall.
Repair Performed	Unplanned restorative intervention; routine prophylactic polishing and minor occlusal adjustment were not classified as repair.
Replacement Performed	Complete removal and replacement of the restoration due to clinical failure.
Debonding	Complete loss of retention. Recorded as a binary restoration-level event and checked for repeated episodes; no restoration debonded more than once.

**Table 4:** Patient-reported outcome measures assessed using a 10-point Visual Analog Scale.

Item	Description
VAS1	Overall satisfaction with the treatment outcome
VAS2	Satisfaction with smile appearance
VAS3	Satisfaction with tooth color
VAS4	Satisfaction with tooth shape and proportions
VAS5	Satisfaction with the natural appearance of the restorations
VAS6	Satisfaction with chewing comfort
VAS7	Satisfaction with speech and phonetics
VAS8	Satisfaction with the integration of the restorations within the smile
VAS9	Satisfaction with gingival appearance around the restorations
VAS10	Willingness to undergo the same treatment again and recommend it to others

**Table 5:** Distribution of restorations according to anatomical region and restorative material.

Region	Lithium Disilicate Restorations	Zirconia Restorations with Biomic LiSi Connect
Maxillary	30	28
Mandibular	20	22
Anterior Region	33	37
Posterior Region	17	13
Total	50	50

**Table 6:** Clinical complications recorded during the three-year follow-up period.

Clinical Outcome	Lithium Disilicate Restorations (Emax) (*n* = 50)	Zirconia Restorations with Biomic LiSi Connect (*n* = 50)	Lithium Disilicate Restorations (Emax) (%) (95% CI)	Zirconia Restorations with Biomic LiSi Connect (%) (95% CI)
Marginal Discoloration	17	19	34 (22.4–47.8)	38 (25.9–51.8)
Secondary Caries	2	3	4 (1.1–13.5)	6 (2.1–16.2)
Hypersensitivity	3	9	6 (2.1–16.2)	18 (9.8–30.8)
Debonding	0	6	0 (0.0–7.1)	12 (5.6–23.8)

**Table 7:** Anatomical distribution of debonding events in zirconia restorations treated with Biomic LiSi Connect.

Region	Number of Debonding Events	Percentage (%)
Anterior Region	5	83.3
Posterior Region	1	16.7
Total	6	100

**Table 8:** Debonding events according to anterior preparation design in zirconia restorations.

Preparation Design	Total Restorations	Debondings	Debonding Rate (%)
Type I	15	3	20.0
Type II	9	2	22.2
Type III	13	0	0.0
Total	37	5	13.5

**Table 9:** Debonding events according to posterior preparation design in zirconia restorations.

Preparation Design	Total Restorations	Debondings	Debonding Rate (%)
Type I	6	0	0.0
Partial occlusal coverage	6	1	16.7
Complete occlusal coverage	1	0	0.0
Total	13	1	7.7

**Table 10:** Three-year restoration survival according to restorative material.

Restorative Material	Total Restorations	Replacements	Survival Without Replacement, % (95% CI)
Lithium Disilicate Restorations	50	0	100 (92.9–100.0)
Zirconia Restorations with Biomic LiSi Connect	50	0	100 (92.9–100.0)
Total	100	0	100 (96.3–100.0)

**Table 11:** Patient-reported outcome scores after three years of clinical follow-up. Visual Analog Scale data are presented as mean values and standard deviations.

VAS Item	Lithium Disilicate Restorations (Mean)	Lithium Disilicate Restorations (SD)	Zirconia Restorations with Biomic LiSi Connect (Mean)	Zirconia Restorations with Biomic LiSi Connect (SD)
VAS1. Overall satisfaction	9.5	0.3	9.6	0.2
VAS2. Smile appearance	9.6	0.3	9.5	0.3
VAS3. Tooth color	9.4	0.4	9.4	0.2
VAS4. Tooth shape and proportions	9.3	0.3	9.6	0.2
VAS5. Natural appearance	9.5	0.2	9.5	0.2
VAS6. Chewing comfort	9.6	0.3	9.7	0.2
VAS7. Speech and phonetics	9.5	0.4	9.6	0.2
VAS8. Integration within the smile	9.7	0.2	9.8	0.2
VAS9. Gingival appearance	9.4	0.4	9.6	0.2
VAS10. Willingness to repeat treatment/recommend treatment	9.7	0.2	9.6	0.3

## Data Availability

The data supporting the results of this study are available from the corresponding authors upon reasonable request, in accordance with institutional policies and ethical regulations.

## Supplementary Materials

The following supporting information can be downloaded at: https://www.mdpi.com/article/10.3390/dj14100621/s1, Table S1, anonymized restoration-level dataset with patient ID, material protocol, tooth, arch, region, preparation design, and debonding status.
